# Exploring mosquito abundance and *Plasmodium* infection through nested-PCR: implications for disease surveillance and control

**DOI:** 10.1038/s41598-024-60662-x

**Published:** 2024-04-30

**Authors:** Haider Abbas, Muhammad S. Sajid, Hafiz M. Rizwan, Urfa B. Tahir, Shahid H. Farooqi, Zeeshan Iqbal, Muhammad A. Malik, Kashaf Yaseen, Mahvish Maqbool, Faiz A. Raza, Mohsin Raza, Dalia Fouad, Farid S. Ataya

**Affiliations:** 1 Department of Parasitology, Faculty of Veterinary Science, University of Agriculture, Faisalabad, 38040 Pakistan; 2 Department of Pathobiology (Parasitology Section), KBCMA College of Veterinary and Animal Sciences, Narowal, Sub-Campus, University of Veterinary and Animal Sciences (UVAS), Lahore, Pakistan; 3 Department of Aquatic Animal Medicine, College of Fisheries, Huazhong Agricultural University, Wuhan, 430070 China; 4 Department of Clinical Sciences (Medicine Section), KBCMA College of Veterinary and Animal Sciences, Narowal, Sub-Campus, University of Veterinary and Animal Sciences (UVAS), Lahore, Pakistan; 5 Department of Animal Sciences (Livestock Section), KBCMA College of Veterinary and Animal Sciences, Narowal, Sub-Campus, University of Veterinary and Animal Sciences (UVAS), Lahore, Pakistan; 6 Institute of Microbiology, University of Agriculture, Faisalabad, 38040 Pakistan; 7 Health Research Institute, National Institute of Health, Research Centre, , King Edward Medical University, Lahore, 54000 Pakistan; 8 Department of Basic Sciences (Physiology Section), KBCMA College of Veterinary and Animal Sciences, Narowal, Sub-Campus, University of Veterinary and Animal Sciences (UVAS), Lahore, Pakistan; 9 Department of Zoology, College of Science, King Saud University, PO Box 22452, Riyadh, 11495 Saudi Arabia; 10 Department of Biochemistry, College of Science, King Saud University, PO Box 2455, Riyadh, 11451 Saudi Arabia

**Keywords:** DNA replication, Genomics

## Abstract

The *Plasmodium* is responsible for malaria which poses a major health threat, globally. This study is based on the estimation of the relative abundance of mosquitoes, and finding out the correlations of meteorological parameters (temperature, humidity and rainfall) with the abundance of mosquitoes. In addition, this study also focused on the use of nested PCR (species-specific nucleotide sequences of 18S rRNA genes) to explore the *Plasmodium* spp. in female *Anopheles*. In the current study, the percentage relative abundance of *Culex* mosquitoes was 57.65% and *Anopheles* 42.34% among the study areas. In addition, the highest number of mosquitoes was found in March in district Mandi Bahauddin at 21 °C (T_max_ = 27, T_min_ = 15) average temperature, 69% average relative humidity and 131 mm rainfall, and these climatic factors were found to affect the abundance of the mosquitoes, directly or indirectly. Molecular analysis showed that overall, 41.3% of the female *Anopheles* pools were positive for genus *Plasmodium*. Among species, the prevalence of *Plasmodium* (*P.*) *vivax* (78.1%) was significantly higher than *P. falciparum* (21.9%). This study will be helpful in the estimation of future risk of mosquito-borne diseases along with population dynamic of mosquitoes to enhance the effectiveness of vector surveillance and control programs.

## Introduction

Mosquitoes are small dipteran insects, which belong to the Culicidae family, and they are cosmopolitan; their larva and pupa grow in different water bodies under favourable conditions^1^. The environmental factors such as temperature, humidity, rainfall, and species habitats are strongly correlated with distribution, survivability and abundance of mosquitoes^2^. The breeding sites/habitats of mosquitoes may vary with seasons and climatic conditions resulting in fluctuation in the population density of mosquitoes^3,4^. They play an important role in the transmission of protozoa, helminths, bacteria and viruses in livestock, pets, wildlife animals and human populations, globally^5–11^.

A total of 3594 species of mosquitoes have been reported worldwide and only 134 species were found in Pakistan^12^. *Anopheles* (*An*.) spp. such as *An. nigerrimus Giles*, *An. peditaeniatus Leicester*, *An. subpictus Grassi *sensu lato, *An. pulcherrimus Theobald*, *An. stephensi Liston s.l.,* and *An. culicifacies Giles s.l.* were found in the irrigated areas of South Punjab, Pakistan^13^. Four species of *Anopheles* and nine species of *Culex* mosquitoes were reported in Murree Hills, Punjab, Pakistan^1^.

*Plasmodium* (Haemosporida: Plasmodiidae), a genus of mosquito-borne protozoan parasites, is responsible for malaria in humans. There are five different species of mosquito-borne *Plasmodium* (*P.*) which infect humans, including *P. falciparum, P. malariae P. vivax, P. ovale* and *P. knowlesi*^14^. Among these species, *P. vivax* causes morbidity, and *P. falciparum* is responsible for mortality. The *vivax* malaria transmission may be found sustained in areas with low transmission intensity due to presence of its dormant forms in hepatic cells^15,16^. Due to complications like algid malaria, kidney malfunction and cerebral malaria, *falciparum* malaria is considered as more fatal^17,18^. The two malarial parasite species, *P. vivax* and *P. falciparum*, are present in Pakistan^19^.

Approximately, 247 million human lives were at risk of *Plasmodium* infection with 619,000 fatalities in 2021 around the globe according to the latest world malaria report^20^. Among the mosquito-borne diseases, malaria, dengue, and chikungunya are the most prevalent in Pakistan. Malaria among the communicable infections is still considered the leading source of morbidity and mortality in Pakistan^19^. About 0.47 million cases of malaria were reported across Pakistan during 2020. From 1960’s to early 80’s, *P. vivax* was considered the most prevalent species of malarial parasite in Pakistan. However, an increasing trend in the prevalence of *P. falciparum* was observed over the last few decades. Recently, *P. vivax* (79.13%) has been the major cause of malaria in humans in Pakistan followed by *falciparum* malaria (16.29%) and mixed infections (3.98%) of these two species^21^. The *P. falciparum* species is rising in Balochistan and Sindh with 108,262 confirmed cases out of 1,484,821 suspected cases across the Sindh province in 2018 which is alarming and 50,000 mortalities occurs every year in Pakistan^19,20^.

Pakistan is among the WHO Eastern Mediterranean regions which is reported as moderate malaria-endemic with 63 million people at high risk and 217 million at moderate risk of malaria. The disease is still present in an endemic state in Pakistan and the 98% proportion of total malaria burden is covered by Pakistan among the seven countries in the region^19,21^. There is dire need of the estimation of future risk of mosquito borne diseases based on mosquito fauna. Therefore, this study is based to estimate the relative abundance of mosquitoes on the conventional microscopy, and finding out the correlation of the meteorological parameters (temperature, humidity and rainfall) with the abundance of the mosquitoes. In addition, this study is also focused on the use of nested PCR to explore the *Plasmodium* spp. in the female *Anopheles* mosquitoes from three climatic zones of Punjab province, Pakistan.

## Results

**Figure 1:**
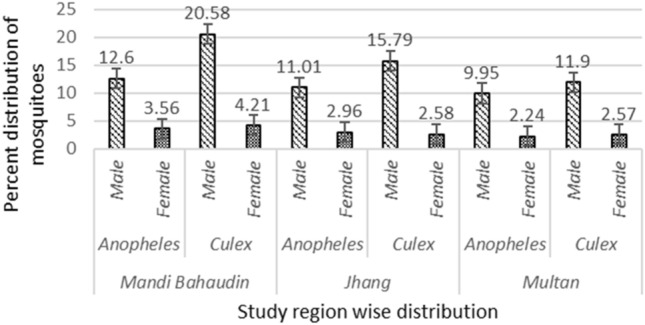
Percent abundance of mosquitoes among different climatic zones of Punjab, Pakistan.

Out of total 27,428 collected mosquitoes, the percentage abundance of *Anopheles* and *Culex* mosquitoes in Mandi Bahauddin, Jhang, and Multan observed from the three climatic zones is given in Fig. 1. In addition, 4435 *Anopheles* and 6800 *Culex* were identified out of total 11,235 mosquitoes collected from three tehsils of district Mandi Bahauddin. About 3833 *Anopheles* and 5042 *Culex* were found out of 8875 mosquitoes from four tehsils of district Jhang. Similarly, 3346 *Anopheles* and 3972 *Culex* were segregated out of total 7318 collected from four tehsils of district Multan. The results have shown that the *Culex* spp. were the most abundant among the mosquitoes in the study areas (Table 1).

**Table 1:** Spatial distribution and abundance of adult mosquitoes.

Study district	Administrative division (Tehsil)	Mosquito genus	Mosquito type	Abundance of mosquitoes (%)
Mandi Bahauddin	Mandi	*Anopheles*	Male	11.2 (1257)
			Female	3.3 (370)
		*Culex*	Male	19.9 (2227)
			Female	3.8 (425)
	Malikwal	*Anopheles*	Male	9.5 (1070)
			Female	2.5 (280)
		*Culex*	Male	14.8 (1665)
			Female	3.5 (391)
	Phalia	*Anopheles*	Male	10.0 (1130)
			Female	2.9 (328)
		*Culex*	Male	15.6 (1753)
			Female	3.0 (339)
Jhang	Jhang	*Anopheles*	Male	9.8 (875)
			Female	2.1 (191)
		*Culex*	Male	13.2 (1172)
			Female	3.2 (285)
	Ahmedpur Sial	*Anopheles*	Male	10.7 (950)
			Female	3.1 (280)
		*Culex*	Male	11.8 (1050)
			Female	2.2 (200)
	Shorkot	*Anopheles*	Male	7.5 (670)
			Female	1.7 (150)
		*Culex*	Male	14.7 (1310)
			Female	1.0 (90)
	Athara Hazari	*Anopheles*	Male	5.9 (525)
			Female	2.2 (192)
		*Culex*	Male	9.0 (800)
			Female	1.5 (135)
Multan	Multan	*Anopheles*	Male	8.1 (600)
			Female	1.5 (110)
		*Culex*	Male	11.1 (815)
			Female	2.7 (198)
	Multan Saddar	*Anopheles*	Male	10.2 (750)
			Female	2.1 (155)
		*Culex*	Male	13.2 (970)
			Female	2.99 (219)
	Shujabad	*Anopheles*	Male	9.1 (670)
			Female	1.6 (120)
		*Culex*	Male	9.7 (710)
			Female	2.6 (192)
	Jalalpur	*Anopheles*	Male	9.7 (710)
			Female	3.1 (231)
		*Culex*	Male	10.5 (770)
			Female	1.3 (98)

**Figure 2:**
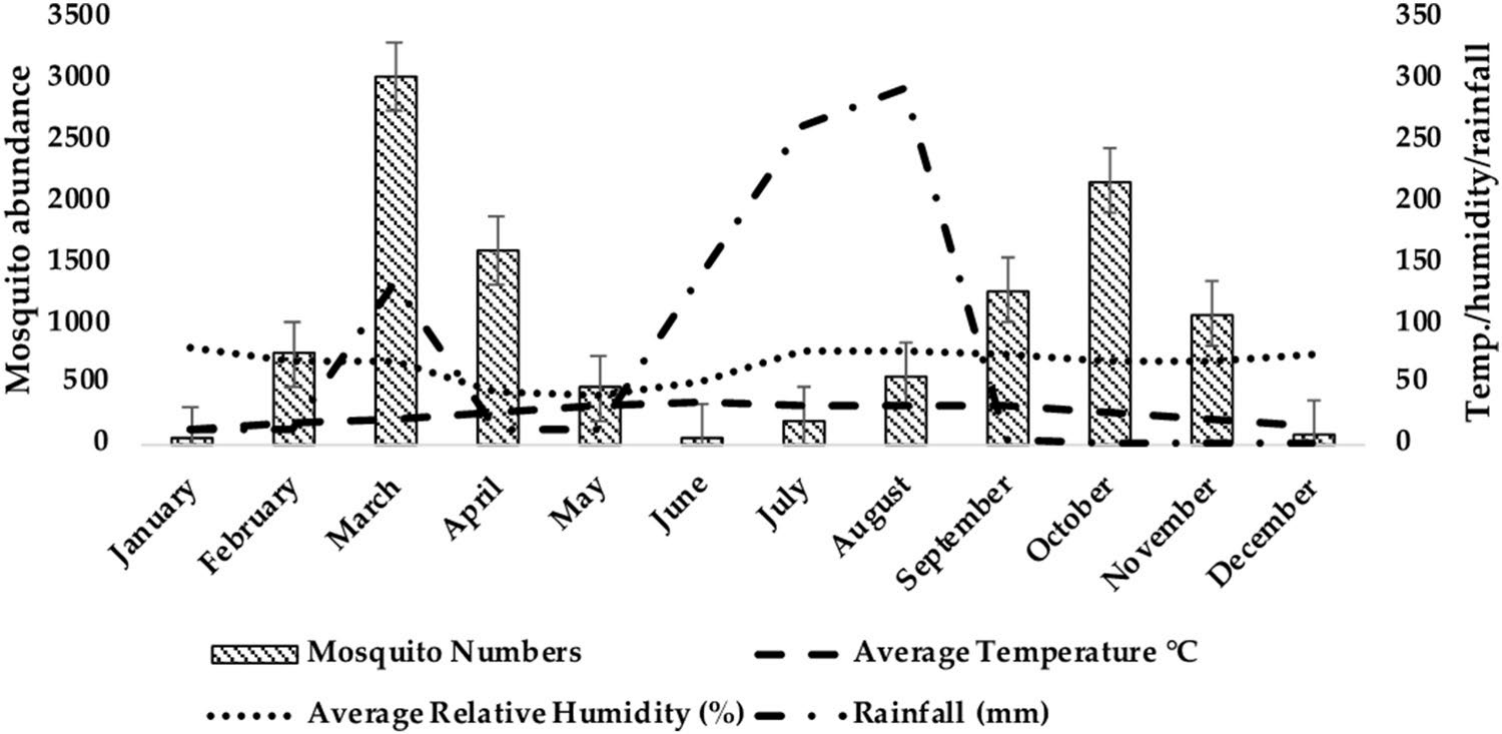
Relationship between climatic factors and adult mosquito seasonal activity in Mandi Bahauddin region.

**Figure 3:**
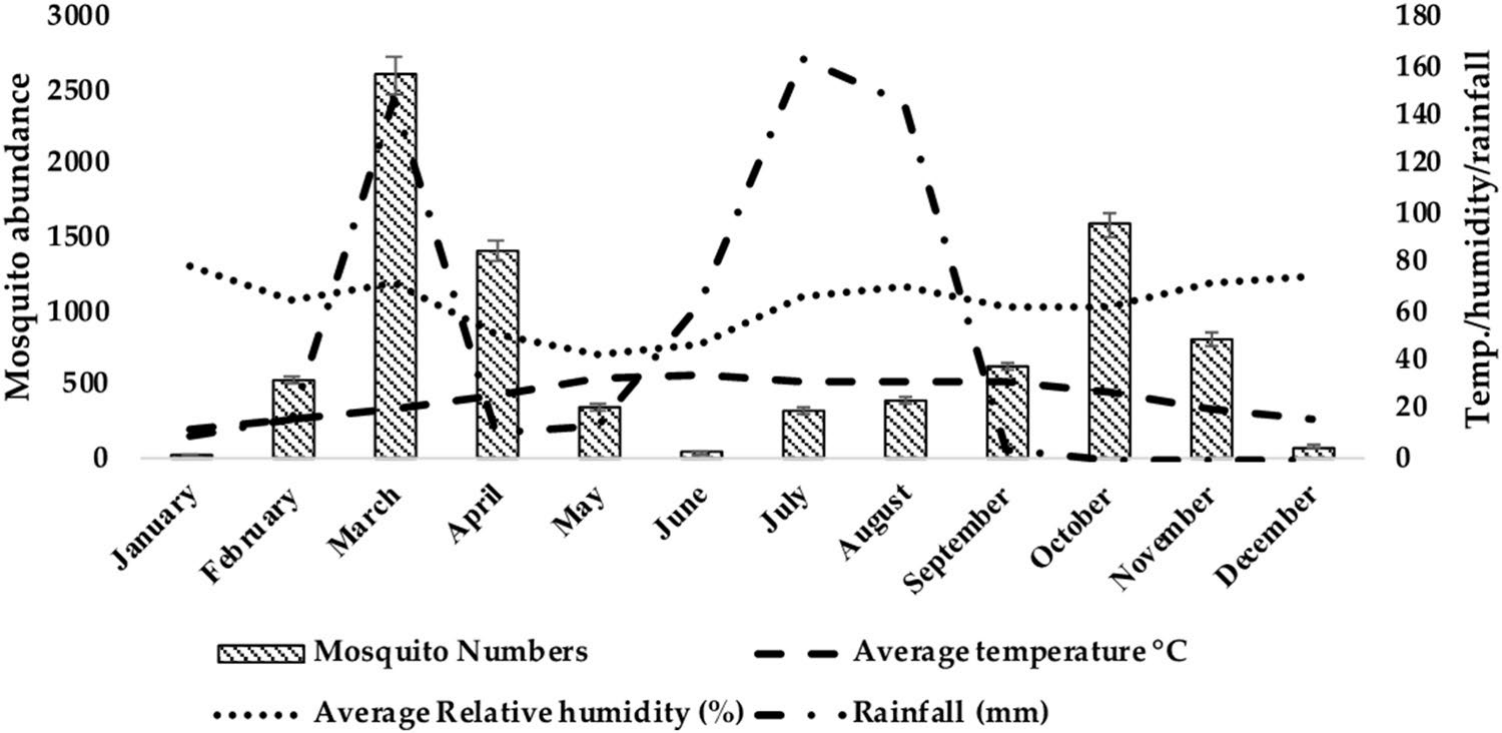
Relationship between climatic factors and adult mosquito seasonal activity in Jhang region.

**Figure 4:**
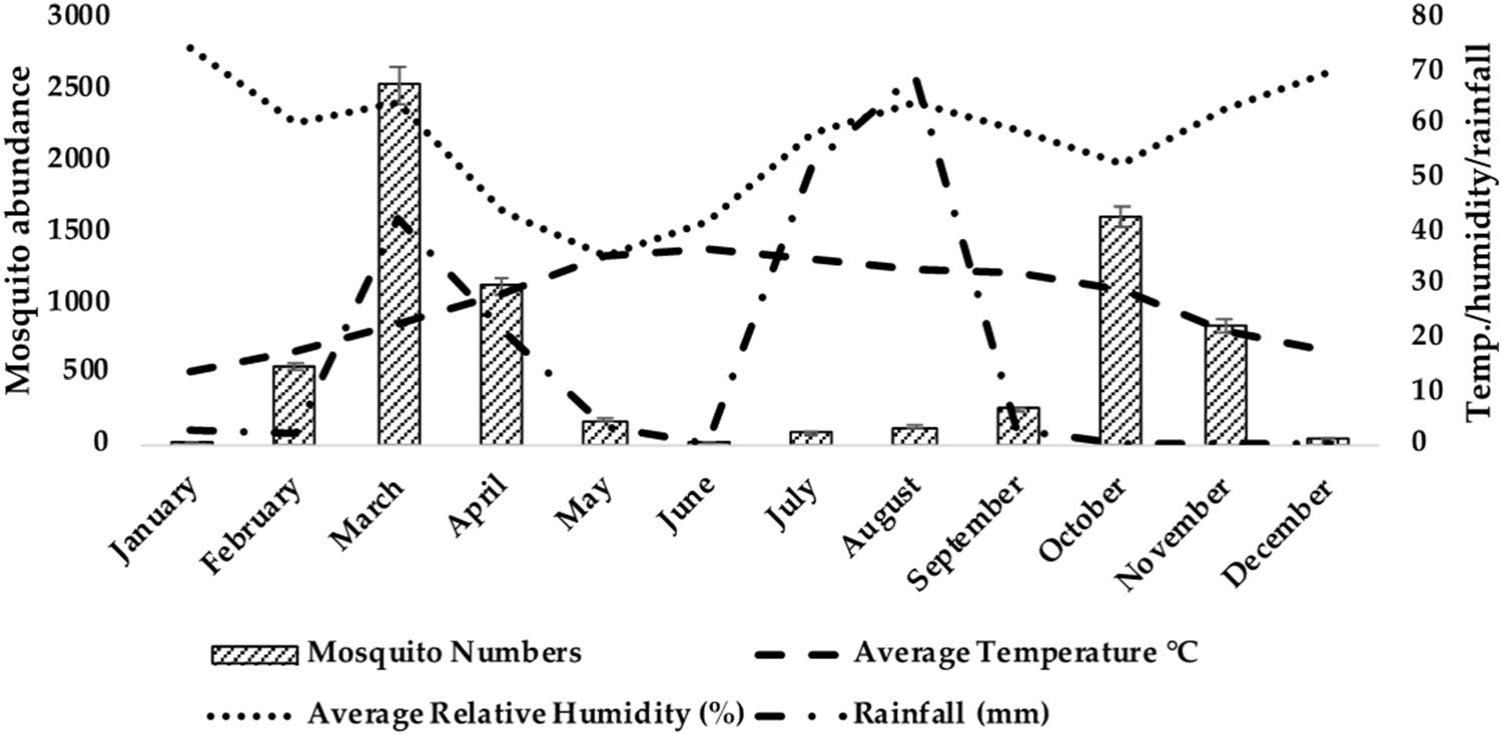
Relationship between climatic factors and adult mosquito seasonal activity in Multan region.

Among all the study districts, the highest average relative humidity (%) was reported in Mandi Bahauddin in January (79.5%), highest temperature (36.45 °C) in Multan in June and maximum rainfall (291 mm) was recorded in August in Mandi Bahauddin. The collective abundance of adult mosquitoes belonging to two genera, *Anopheles* and *Culex* was found to be higher during March, April and October in the study areas (Figs. 2, 3, and 4). The Table 2 shows the output of the Pearson’s correlation between abundance of mosquitoes and monthly average temperature (°C), average relative humidity (%) and rainfall (mm) for three study areas. It is evident that in Mandi Bahauddin, average temperature showed weak positive correlation (0.015) with *Anopheles*, and weak negative correlation with *Culex* (− 0.021). Similarly, average relative humidity had weak negative correlation (− 0.045) with *Anopheles* and *Culex* (− 0.065). However, rainfall was found to be negatively correlated to *Anopheles* (− 0.201) and *Culex* (− 0.106). In the district Jhang, the average temperature was weakly and negatively correlated to the *Anopheles* (− 0.084) and *Culex* (− 0.032), while the average relative humidity was positively and weakly correlated to the *Anopheles.* In addition, rainfall was recorded weakly and positively correlated to the *Anopheles* (0.073) and *Culex* (0.293). Similarly, In the district Multan, average temperature showed negative and weak correlation with the *Anopheles* (− 0.150) and *Culex* (− 0.184). However, average relative humidity was weakly and negatively correlated to the *Anopheles* (− 0.026) while weakly and positively correlated to *Culex* (0.018). Lastly, rainfall was found to be weakly and positively correlated to *Anopheles* (0.040) and *Culex* (0.174). However, the relationship of environmental parameters (relative humidity, average temperature and rainfall) with the abundance of mosquitoes in districts Mandi Bahauddin, Jhang and Multan was observed as non-significant (*p* > 0.05). The highest number of mosquitoes was found in March in district Mandi Bahauddin at 21 °C (T_max_ = 27, T_min_ = 15) average temperature, average relative humidity 69% and rainfall 131 mm (Fig. 2).

**Table 2:** Pearson’s correlation coefficient (r) between mosquito abundance and climatic factors in study areas.

	*Anopheles*		*Culex*	
	r-value	*p* value	r-value	*p* value
Mandi Bahauddin				
Average temperature (°C)	0.015	0.964	− 0.021	0.948
Average relative humidity (%)	− 0.045	0.890	− 0.065	0.841
Rainfall (mm)	− 0.201	0.532	− 0.106	0.742
Jhang				
Average temperature (°C)	− 0.084	0.795	− 0.032	0.922
Average relative humidity (%)	0.004	0.991	0.105	0.745
Rainfall (mm)	0.073	0.822	0.293	0.355
Multan				
Average temperature (°C)	− 0.150	0.642	− 0.184	0.567
Average relative humidity (%)	− 0.026	0.936	0.018	0.955
Rainfall (mm)	0.040	0.902	0.174	0.589

**Figure 5:**
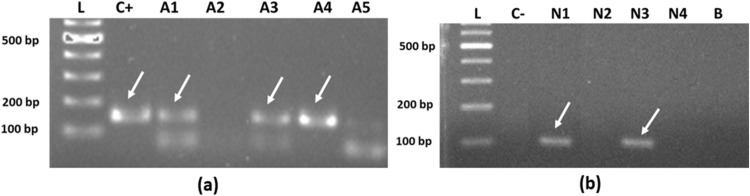
Molecular identification of *Plasmodium* spp.: (**a**) Gel representation of 160 bp cDNA fragments of first round amplification; (**b**) 110 bp cDNA fragments of second round amplification. [Lane L: 100 base pair (bp) plus DNA ladder, Lane C + : positive control, Lane A1–A5: female *Anopheles* mosquito PCR products of first reaction of nest-PCR, Lane N1–N4: PCR products of *Plasmodium* species specific primers (for *vivax* [N1], *falciparum* [N2]*, ovale* [N3] and *malariae* [N4]), Lane C-: negative control, Lane B: blank].

The first round PCR targeted 18S rRNA gene of *Plasmodium* genus revealed a band of 160 bp. The band revealed at 160 bp as shown in Fig. 5a was considered as positive result for the presence of genus *Plasmodium*. The overall prevalence of *Plasmodium* in the female *Anopheles* mosquitoes of the study areas was found to be 41.3% (64/155 pools) with a higher *Plasmodium* distribution in district Mandi Bahauddin followed by Multan and Jhang (Table 3). The administrative division-wise (tehsil-wise) prevalence of *Plasmodium* among vectors of the study districts was highest in the Mandi Bahauddin (66.6%), followed by Malikwal (44.4%), Ahmedpur Sial (44.4%), Multan (42.8%), Multan Saddar (40%), Jalalpur (40%), Phalia (38.1%), Jhang (37.5%), Athara Hazari (25%), Shujabad (25%), and Shorkot (22.7%). A non-significant (*p* > 0.05) difference among prevalence of *Plasmodium* spp. in *Anopheles* mosquitoes was observed in various study administrative divisions.

**Table 3:** Prevalence of genus *Plasmodium* and species of *Plasmodium* in vector population of the study areas.

Study area	gDNA pools screened	*Plasmodium* positive pools (%)	*P. vivax* positive pools (%)	*P. falciparum* positive pools (%)
Mandi Bahauddin	63	32 (50.8)	24 (75)	8 (25)
Jhang	52	17 (32.7)	15 (88.2)	2 (11.8)
Multan	40	15 (37.5)	11 (73.3)	4 (26.6)
Total	155	64 (41.3)	50 (78.1)	14 (21.9)

For the 2nd round PCR (species specific), the desired band revealed at 110 bp indicating the species of *Plasmodium*. Among *Plasmodium* species, *P. vivax* and *P. falciparum* produced the desired band as shown in Fig. 5b. A total of 50 out of 64 positive pools were found positive for *P. vivax* (78.1%), and 14 pools for *P. falciparum* (21.9%)*.* No *P. ovale* and *P. malariae* were found in the present study.

## Discussion

Mosquitoes have medical importance as they act as vectors of various disease agents affecting millions of humans, annually. *Aedes*, *Anopheles*, *Culex*, *Mansonia* and *Culiseta* are the mosquito genera which may act as vectors^27–29^. The abundance of *Anopheles* mosquitoes was low as comparison to *Culex* spp. which could be due to various factors including sampling approach and better vector control strategies in urban areas with proper sanitation. The *Culex* mosquitoes attracts more towards livestock and human population than any other mosquitoes, therefore this might be the one reason of higher abundance^30,31^. In different studies, *Culex* were also found in abundance than other mosquitoes in Kenya^32^, in North Kent, UK^33^, and in Iran^31^. The watery habitat of rice field is more suitable for the development of *Culex* larvae and can be a factor for higher *Culex* abundance^34,35^.

These results showed that mosquitoes in Mandi Bahauddin were present abundantly as compared to Jhang and Multan at average relative humidity 50–80%, average temperature 15–30 °C and rainfall 130 mm. This might be due to the change in the climatic conditions of these three districts as Multan is situated in North zone of Punjab and Jhang in Central zone where climatic conditions are slightly different as compared to Mandi Bahauddin where climatic conditions are favorable for survival of mosquitoes. Numerous studies^36–40^ support the findings here that also show association between climatic factors and mosquito seasonal activity. Moreover, these results are also in agreement with other studies which showed that on the increase in temperature, the abundance of mosquitoes reduced due to higher metabolic rate and lower respirations rate leading to increase in mortality, and 15–28 °C temperature range found to be more favorable for the survivability and proper growth of mosquito fauna^41,42^. It has been presented in studies that ovipositing, larval growth and mosquito activity increased on high humidity levels^43,44^. In some studies, 44–69% RH range has been reported as more favourable for mosquito abundance^42,44^. The average rainfall has positive impact on mosquito abundance due to increase in suitable breeding sites^44^. The precipitation range 80–120 mm was reported suitable for mosquito population in a study^37^. The population density, diversity and distribution of mosquito fauna are strongly affected by the fluctuations in the climatic (including average relative humidity, average temperature and rainfall) and ecological conditions^4,45^.

In the present study, through nested PCR, *P. vivax* and *P. falciparum* among *Plasmodium* species have been identified in the anopheline vectors, which is in agreement with the study of Soomro et al*.*^46^. The WHO reports on malaria in Pakistan state that *P. vivax* and *P. falciparum* are the only dominant malaria-causing species in Pakistan^47^, and similarly, in our study, only two species, *P. vivax and P. falciparum* were identified in the study regions.

A similar study conducted in Faisalabad (Pakistan) reported the prevalence of the genus *Plasmodium* about 46% (14/30 pools) *in Anopheles* mosquitoes, along with *P. falciparum* and *P. vivax*^25^. The infection rate of 0.46% (566/123,286) for *Plasmodium* species was reported in the anopheline vectors in a systematic review and meta-analysis conducted in Thailand^48^. A study in Ubon Ratchathani Province, Thailand, detected *P. vivax*-210 and *P. vivax*-247 in the pooled *Anopheles* (1/29 pools; 3.45%) through ELISA and nested PCR^49^. Through the nested PCR technique, *P. vivax* was identified in DNA extracts (3/109; 2.75%) of *Anopheles* mosquitoes from the two provinces in Thailand^50^.

All the following studies on malaria prevalence in Pakistan have been conducted on blood samples, either through conventional microscopy or molecular techniques. A slide positivity rate of 35% was reported for the *Plasmodium* genus along with two species of *P. vivax* (66.8%) and *P. falciparum* (30.7%) in Quetta, Pakistan^51^. Similarly, a study conducted in Punjab province (including five cities) presented *P. vivax* (73%), *P. falciparum* (18%), and 5% of mixed infections of these two species^52^. Moreover, a higher prevalence of *P. vivax* in comparison to *P. falciparum* (99.4% vs. 0.53%) was reported in Lal Qilla Lower Dir, Khyber Pakhtunkhwa (KPK), Pakistan^53^. In Multan, 60.5% *P. vivax* and 37.2% *P. falciparum* were reported^54^. In Northern and Southern Punjab (10 cities), *P. vivax* (53.4%), *P. falciparum* (18.7%), and mixed infections of these two species (12.7%) were reported based on molecular analysis of blood samples. In another study of 1,127 malaria-susceptible patients in Pakistan's KPK province, molecular analysis (nPCR) revealed an overall prevalence of 38% of the genus *Plasmodium*, with 87% *P. vivax* and 13% *P. falciparum*^55^. Furthermore, based on nPCR, *P. vivax* (81.1%), *P. falciparum* (13.8%), and mixed infections of these two species (4.9%) were reported^56^. The study conducted in tribal areas revealed a higher prevalence of *P*. *vivax* than* P*. *falciparum* in March, while a contrast was observed in October^57^.

In contrast, the study conducted in Malakand district found a *Plasmodium* prevalence of 26.7%^58^. Likewise, in Karachi, Pakistan, in 2002, a higher prevalence of *P. falciparum* (65%) as compared to *P. vivax* (35%) was reported among the malaria-suspected children^59^. A high percentage of *P. falciparum* to *P. vivax* (88.5% vs. 9%) was reported in another study conducted in Karachi, Pakistan^60^. Based on 175 suspected malarias patients at the Khyber Teaching Hospital, Peshawar, Pakistan, from 2011 to 2014, *P. falciparum* was found in 71.43% of samples^25^. A similar pattern of prevalence of *P. falciparum* was observed in the southwestern region of Pakistan. For example, it was 58.9% (*P. falciparum*) and 41% (*P. vivax*) in the Larkana district^61^. The infection rates of *P. falciparum* and *P. vivax* were reported at 52.8% and 47.1% in another study in the Larkana district, respectively^46^. Furthermore, in Hyderabad, it was 52.5% and 46.5% in one study^62^, while in another research study, it was 47% and 53%^63^. Similarly, in Quetta, Dera Ismail Khan, and Karachi, a higher prevalence of *P. falciparum* than *P. vivax* (65%, 10.7%, and 58.1%) was reported, respectively^59,64,65^.

Malaria transmission is not always stable but rather found to be unstable, with *P. falciparum* transmission peaking between August and December while *P. vivax* transmission gains its peak level in two phases (first, from June to September, and second, from April to June) in Pakistan^13,66,67^. Therefore, the non-probability sampling and/or collection of mosquitoes might be related to a higher *vivax* prevalence as compared to *falciparum,* and these findings are also in agreement with those of Shahwani et al.^68^. In addition, the higher prevalence of malaria might be due to the development of resistance to chloroquine^69^. and ineffective vector control strategies in the area^70,71^. The development of resistance against most antimalarial drugs has been reported in *Plasmodium* spp.^72^ Moreover, the socio-demographic factors affecting the disease dynamics include exponential population growth, housing condition, occupation, awareness about health care services, urbanization, age, gender, excessive monsoon rains, and strategies used to avoid mosquito bites^27,66,73^.

The molecular tools have been proven more sensitive and accurate for the diagnosis of malarial infections than the conventional (classic) methods. The infection of *P. vivax* and *P. falciparum* through this technique has also been identified in a similar study in the Caribbean^74^. However, the nested PCR protocol used in this study was optimized using the same primers and conditions reported by Han et al.^24^ and Hayat et al.^25^, both of which detected *Plasmodium* infection in blood samples and in female *Anopheles,* respectively. In our study, there were no findings for *P. ovale* or *P. malariae,* which is aligned with a study conducted in Multan^75^ and Faisalabad, Pakistan^25^ and with the WHO World Malaria Report-2019. Moreover, no clinical cases in humans caused by *P. ovale* or *P. malariae* have been reported in Pakistan so far^19,21^.

Along with microscopy, immunochromatographic tests, and a rapid diagnostic test (RDT) have been used for the confirmation of antigens using monoclonal antibodies. On the other hand, these procedures are not sensitive or specific for the diagnosis of certain species of *Plasmodium*^76^. These RDTs and conventional techniques are quick, uncomplicated, and simple in interpretation, but they have the potential only for the identification of *P. falciparum* and *P. vivax*. Molecular assays that are more sensitive and specific than RDTs or conventional microscopy include nested multiplex-PCR, semi-nested-PCR, and nested-PCR^77^.

In conclusion, nested-PCR assay resulted in the identification of *P. vivax* and *P. falciparum* in this study. The molecular techniques, being costly, are regarded as less useful in routine clinical diagnosis, but their potential for specific *Plasmodium* spp. identification in blood and mosquito vectors can help in early surveillance, ultimately reducing the disease threat. Further parallel studies are recommended to confirm the different species of *Plasmodium* in mosquitoes as well as malaria-susceptible humans to ensure the presence of different *Plasmodium* species in a specific area in the future. In addition, climate change has resulted in variations in the pattern of precipitation (rainfall) and temperature, which affect the mosquito population directly or indirectly. However, climatic factors (relative humidity, temperature, and rainfall) have shown correlation with the numbers of mosquitoes, but their effect is complex, therefore, the influence of weather variables on mosquito vector behavior should be monitored through tools like the Global Positioning System (GPS) to enhance the understanding of these correlations. Moreover, the findings of this study provide a way forward to implement strict vector control measures to reduce the chances of vector-borne diseases in humans and animals.

## Methods

### Study areas

**Figure 6:**
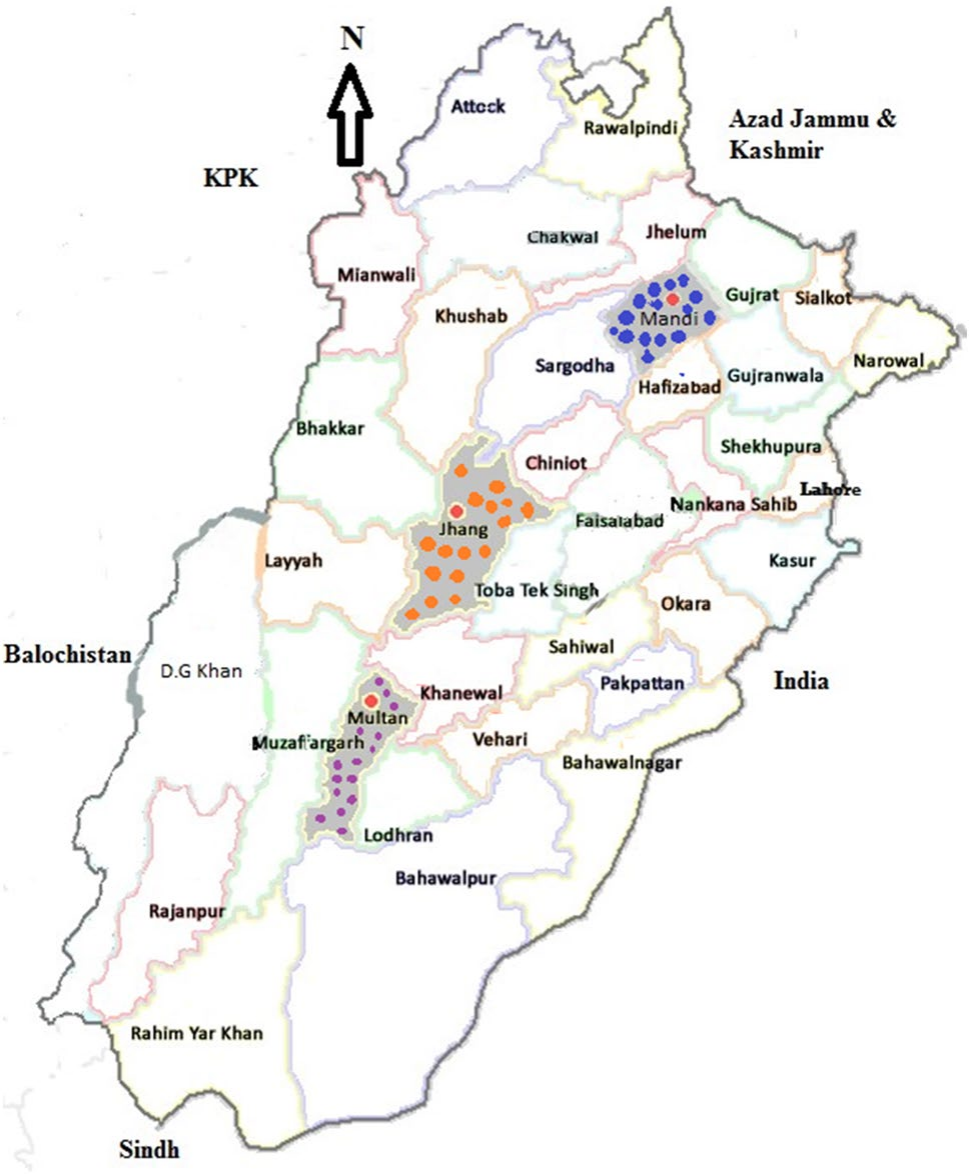
Physical map of Punjab province representing the sampling sites in study areas [Source: Paint App in Microsoft Windows Version 22H2 (OS Build 19,045.4046)].

Three districts Mandi Bahauddin (73° 36′ to 73° 37′ E and 30° 8′ to 32° 40′ N), Jhang (71.37-to-73.13° E and 30.37-to-31.59° N) and Multan (71° 28′ 11′′ E and 30° 11′ 52′′ N) situated in South, Central and North climatic zones of Punjab were selected for sampling of mosquitoes (Fig. 6). The district Mandi Bahauddin is consisting of three tehsils (administrative divisions) including Mandi Bahauddin, Malikwal, and Phalia; district Jhang consisting of four tehsils including Ahmedpur Sial, Athara Hazari, Jhang and Shorkot, and district Multan consisting of four tehsils like Multan, Multan Saddar, Jalalpur, and Shujabad.

Mandi Bahauddin district has cold winter and hot summer which depict moderate climate of this district with average temperature range 3–48 °C, average rainfall 9.19–166.73 mm and relative humidity 22–51%. District Jhang climate scenarios include hot and dry summer season while winter is dry and cold with annual average temperature range 19.7–41.1 °C, average rainfall 4–76 mm and relative humidity 36–66%. While Multan district topography includes loose, flat, unconsolidated soil or sediments which are very suitable for agriculture. Climate of Multan is arid representing clement winters and scorching summers with max temperature recorded 52 °C and minimum temperature − 1 °C, average rainfall 7–55 mm and relative humidity 35–60%.

### Entomological sampling, data collection and processing

Mosquitoes were collected from areas of human population, hospitals, livestock farms, poor households, lavatories, standing water ponds and sewage contaminated fields using two methods at each location: insect collecting nets and light baited traps (DP. LED Light, Guangzhou, China) during year 2022. Monthly mosquito collection was performed in three districts with a total of 45 locations (15 locations per district). Collection was done for 3 consecutive nights per month at each location between 17:00 and 7:00 h and preserved in 70% ethanol. Mosquitoes were brought to the Molecular Parasitology Laboratory, University of Agriculture, Faisalabad (UAF), Pakistan for morphological identification. Mosquitoes were identified under stereoscopic microscope using standard taxonomic keys provided by Soulsby^22^ and Becker et al.^23^. The data related to meteorological factors (temperature, humidity and rainfall) of the selected districts for the study duration was obtained from Pakistan Meteorological Department (Regional Meteorological Center, Lahore) (https://rmcpunjab.pmd.gov.pk/www/index.php). Then, the correlation of abundance of the mosquitoes with the meteorological factors (temperature, humidity and rainfall) was analyzed.

The female *Anopheles* were procured, identified and separated from the rest of mosquitoes for molecular analysis. Female *Anopheles* were pooled into 1.5 mL Eppendorf tubes after microscopic identification (15 females per pool) and total 155 pools of mosquitoes were made from the February-June collection and from the August-December collection. Then, gDNA was extracted using EZ-10 Spin Column Genomic DNA Miniprep Kit, Animal Samples (CAT. #: BS427; Bio Basic Canada Inc.) following the given protocols. Quantification and quality assessment of the extracted DNA were performed by Nanodrop spectrophotometer 2000 @A260/A280 purity ratio (ThermoFisher Scientific, USA).

### Nested PCR

The extracted DNA of *Anopheles* were subjected to amplification of 18S rRNA genes (species-specific nucleotide sequences) using nested PCR capable of detecting individual species including *P. falciparum, P. vivax, P. ovale* and *P. malariae* and mix infection as described by Han et al.^24^ and Hayat et al.^25^. The nested PCR consisted of two reactions and during the first-round amplification, 25 μL reaction was prepared containing 12.5 μL of Red Dye PCR (2x) Master Mix (GeNeiTM consisting of 400 μM of each DNTP, 0.6 units of Taq DNA Polymerase, Tris buffer pH 8.5 and 3 mM of MgCl2), 1 μL (0.5 μM) each of *Plasmodium* universal forward (P1: 5′-ACGATCAGATACCGTCGTAATCTT-3′) and reverse (P2: 5′-GAACCCAAAGACTTTGATTTCTCAT-3′) primers, 3 μL of sample DNA and 7.5 μL nuclease free water. The DNA amplification protocol used for the first round of the nested PCR was initial denaturation at 94 °C for 10 min followed in order by 35 cycles of 92 °C for 30 s, 60 °C for 1.5 min, 72 °C for 1 min and final extension at 72 °C for 5 min. The 2nd round PCR was targeted on species specific gene fragments of various *Plasmodium* genus members. The species-specific primers were utilized in 2nd round for each *Plasmodium* spp. The second round of amplification was same as that of first round except the following; 1 μL of each species-specific reverse primer in a separate PCR tube (*P. malariae*: 5′-GGAAGCTATCTAAAAGAAACACTCATAT-3′, *P. ovale*: 5′-ACTGAAGGAAGCAATCTAAGAAATTT-3′, *P. falciparum*: 5′-CAATCTAAAAGTCACCTCGAAAGATG-3′, *P. vivax*: 5′-CAATCTAAGAATAAACTCCGAAGAGAAA-3′), 1 μL of 20 folds diluted first reaction DNA with 20 cycles of amplification (using same thermocycling temperatures as first round). The amplified products were visualized on ethidium bromide stained 1.5% agarose gel under Gel Doc™ XR + Gel Documentation System (Bio-Rad, USA). Moreover, the extraction of DNA, preparation of PCR reactions and replication of DNA were performed by different sets of pipettes and in clean and specific work areas to avoid cross-contamination. The positive bands were observed against a 100 bp plus molecular weight markers. The positive controls (DNA templates of *Plasmodium*) were provided by PMRC Research Center, Punjab Medical College [PMC], Faisalabad-38000, Pakistan, and the negative controls lacking template DNA were run on the gel as reference.

### Statistical analyses

The relationship of abundance of mosquitoes with climatic parameters (temperature, relative humidity and rainfall) was statistically analyzed on monthly basis through Pearson’s correlation coefficient (r) to find out the direction and strength of the linear correlation^4,26^. However, the prevalence of *Plasmodium* spp. in the vector population of the study areas was statistically investigated through chi-square test of independence in contingency tables using R software (https://www.r-project.org/). Variables were considered statistically significant at *p* < 0.05.

## Data Availability

The data used to support the results of this study are included within the article.
